# The Translation, Validation and Cultural Adaptation of Questionnaires Assessing the Quality of Life and Fatigue among Patients with Sjogren’s Syndrome for the Romanian Context

**DOI:** 10.3390/clinpract13060137

**Published:** 2023-12-05

**Authors:** Denise-Ani Mardale, Daniela Opriș-Belinski, Violeta Bojincă, Mihai Bojincă, Emilia Păsăran, Ioana Săulescu, Florian Berghea, Andra Bălănescu

**Affiliations:** 1 Faculty of Medicine, ‘Carol Davila’ University of Medicine and Pharmacy, 050474 Bucharest, Romania; daniela.opris@umfcd.ro (D.O.-B.); violeta.bojinca@umfcd.ro (V.B.); mihai.bojinca@umfcd.ro (M.B.); emilia-daniela.pasaran@drd.umfcd.ro (E.P.); ioana.saulescu@umfcd.ro (I.S.); florian.berghea@umfcd.ro (F.B.); andra.balanescu@umfcd.ro (A.B.); 2 Department of Internal Medicine and Rheumatology, ‘Sf. Maria’ Clinical Hospital, 011192 Bucharest, Romania; 3 Department of Internal Medicine and Rheumatology, ‘Dr. Ion Cantacuzino’ Hospital, 020475 Bucharest, Romania

**Keywords:** Sjogren’s syndrome, fatigue, quality of life, translation, questionnaire

## Abstract

About 70% of patients with Sjogren’s syndrome suffer from fatigue, and for a large proportion of patients, it is one of the most noticeable manifestations leading to disability. To date, no study has been conducted in Romania to determine the quality of life of patients and the impact of fatigue in patients with Sjogren’s syndrome. The present work proposes the translation, cultural adaptation, and validation of two questionnaires for the Romanian context, namely the ‘Profile of Fatigue and Discomfort–Sicca Symptoms Inventory’ (PROFAD-SSI-SF) and ‘Primary Sjogren’s Syndrome—Quality of Life’ (PSS-QoL), whose purpose is to assess quality of life and fatigue in patients with Sjogren’s syndrome. These two questionnaires were administered to 52 patients with Sjogren’s syndrome diagnosed according to the 2016 ACR-EULAR criteria. Subsequently, the conceptual, semantic, and operational analyses of the data were performed with the aim of cultural adaptation. The data obtained were statistically analyzed using indices of measurement accuracy such as internal consistency. Based on statistical analyses, this pilot study shows that the Romanian versions of the PROFAD-SSI and PSS-QoL questionnaires are as reliable as their English counterparts.

## 1. Introduction

Primary Sjogren’s syndrome is a heterogeneous autoimmune disease characterized by the hypofunction of the exocrine glands, particularly the lacrimal and salivary glands, due to a lymphocytic infiltrate, but may also be associated with systemic manifestations [1]. This condition affects patients physically, psychologically, and socially and impacts their quality of life, with fatigue playing a central role [2,3,4,5].

We emphasize that patients are best placed to assess the impact of treatment on pain, function, symptoms, and quality of life. Patient response outcomes (PROs) also play a crucial role in patient-centered care, and data collection ensures quality and safety [6]. The benefits of using PROs extend to the individual level and support collaborative decision making, participation, and self-management [7].

A literature review of PROs describes the main reasons for their use.

1. The Chalder Fatigue Questionnaire (CFQ): This was developed to assess the severity of fatigue in hospital and community populations. In 2015, a study was published on the utility of this questionnaire in Sjogren’s syndrome. The study was carried out in the UK and included 150 patients with primary Sjogren’s syndrome (pSS). The conclusion showed that cognitive impairment is more common in pSS than in the general population and that anxiety is a predictor of cognitive failure in pSS [8].
2. Functional Assessment of Chronic Illness Therapy (Fatigue) (FACIT-F): This was developed in 1997 to measure fatigue in oncology. However, it has subsequently been used to assess chronic illness, and more recently, Natasha Griffiths et al. have demonstrated the content validity of this questionnaire in pSS [9].
3. The Multidimensional Fatigue Inventory (MFI): Published in 1995, this assessment tool was developed to measure cancer fatigue and chronic fatigue syndrome. In 1998, it was used to assess fatigue in pSS patients and is based on 20 questions covering different dimensions of fatigue such as general, physical, and mental fatigue; a reduction in motivation; and a reduction in activity. A study published in the Netherlands demonstrated increased levels of fatigue in patients with primary SS compared to healthy controls [10]. In 2008, an article comparing the Profile of Fatigue (ProF) and the Multidimensional Fatigue Inventory (MFI) was published, with good results [11].
4. Short-Form-36 Vitality Subscale (SF-36 VT): This is a multidimensional, general, patient-reported measure of health status and contains subscales for eight domains. It was first published in 1992 [12] and has been updated several times. This versatile instrument, SF-36, has applications in a number of areas, including health research, clinical practice, and health policy development. It was developed by R. J. Barry et al., who published an article focused on the SF-36, which played a crucial role in the development of the Profile of Fatigue and Discomfort–Sicca Symptoms Inventory (PROFAD-SSI), a novel instrument for assessing fatigue in people with Sjogren’s syndrome. This tool was discussed in detail in the sequel to this article, focusing on patients with Sjogren’s syndrome [13].
5. Visual Analog Scale (VAS): This is a unidimensional measure used to quantify the severity or intensity of fatigue. Over time, it has been used in several clinical trials with other scales to assess different aspects of pSS patients. For example, in 2011, Karstein Haldorsen et al. published the results of a study conducted on 122 patients with Sjogren’s syndrome who were assessed using different questionnaires: Fatigue Severity Scale (FSS), Fatigue Visual Analog Scale (VAS), Functional Assessment of Chronic Illness Therapy (Fatigue) (FACIT-F), and Medical Outcome Study Short-Form-36 (SF-36). The study showed that fatigue remained unchanged over time, regardless of the assessment method used [14].
6. Profile of Fatigue and Discomfort–Sicca Symptoms Inventory—Short Form (PROFAD-SSI-SF): In 2003, Bowman and colleagues pioneered the development of the SSI questionnaire [15] designed to assess Sicca symptoms. The following year, in 2004, they began creating the PROFAD questionnaire, tailored to assess fatigue and discomfort in individuals diagnosed with Sjogren’s syndrome. In total, 130 patients participated in this endeavor [16]. Following the successful development and validation of this instrument, known as the Profile of Fatigue and Discomfort–Sicca Symptoms Inventory (PROFAD-SSI), comprising 64 questions spread across eight domains, a scientific article was published in 2009 recommending the use of its shortened 19-question counterpart, known as the Profile of Fatigue and Discomfort–Sicca Symptoms Inventory—Short Form (PROFAD-SSI-SF). In the same year, Bowman S.J. et al. validated the Sicca Symptom Inventory, and thus PROFAD-SSI was born [17].
7. Primary Sjogren’s Syndrome—Quality of Life (PSS-QoL): This questionnaire was developed in 2018, and it is the first specific instrument to assess health-related quality of life in patients with Sjogren’s syndrome. It was applied to 123 patients, and the results showed that lower perceived dryness was associated with higher immunological activity as determined by increased levels of Ig G and RF IgA, while patients with only subjective signs of dryness had lower immunological activity [18,19].

To ensure standardized patient assessment and facilitate cross-border collaboration, it is essential to validate these scales in as many countries as possible. Therefore, with the aim of gaining a deeper understanding of the disease and assessing its clinical and psychosocial impact, we decided to translate, culturally adapt, and validate two questionnaires for the Romanian context: Profile of Fatigue and Discomfort–Sicca Symptoms Inventory and Primary Sjogren’s Syndrome—Quality of Life [17,18].

In 2018, Romão VC et al. published an article analyzing Sjogren’s syndrome in terms of the availability of treatment interventions applicable in clinical practice, noting the lack of diagnostic and treatment guidelines. Thus, among the first signs of the disease, we find fatigue, pain, and cognitive dysfunction, elements that are often overlooked by doctors but which contribute significantly to the patient’s disability. It is a great challenge to understand the real impact of these symptoms on patients’ lives and to comprehend the pathogenesis (e.g., whether depression arises from intense disease activity or is related to fatigue) [20].

### 1.1. Assessment of Fatigue in Patients with Primary Sjogren’s Syndrome: Data from the Literature

Sjogren’s syndrome affects patients not only in terms of multiorgan damage but also from a psychosocial perspective, which is an important factor [21]. Thus, a new method of studying the disease was proposed by placing each manifestation in specific categories according to pathophysiology, thus enabling the development of specific instruments for assessing the severity [6].

The evaluation of the disease’s impact has been the subject of several studies. EULAR developed scales for assessing the disease activity from the perspective of both healthcare providers (ESSDAI) and patients (ESSPRI). There were also studies that used established SF-36-type questionnaires, which identified fatigue as a significant issue among patients, affecting approximately 75% of cases [7,22,23,24]. Patients with primary Sjogren’s syndrome have a lower quality of life than the general population, which is influenced by factors such as fatigue, pain, anxiety or depression, dental problems, and disease activity. This was demonstrated by a study conducted in China, which included 185 patients with primary Sjogren’s syndrome and evaluated them using the SF-36 questionnaire [25].

The interest in Sjogren’s syndrome is not only reflected in the development of questionnaires to assess symptoms and quality of life but there is also a strong emphasis on early diagnosis. Thus, in 2022, a screening questionnaire for patients with Sjogren’s syndrome, consisting of five questions, was developed in the School of Medicine at the University of Pennsylvania. Its use in clinical practice can reduce the delay in establishing a diagnosis [26].

The quality of life in pSS was also investigated in a group of 61 patients assessed with a questionnaire called OHIP-14 (Oral Health Impact Profile-14), which was developed by dentists in Spain. The responses to this questionnaire showed a positive correlation with both clinical manifestations, such as xerostomia, and a decrease in quality of life [27].

The need for a multidisciplinary approach to patients with Sjogren’s syndrome is demonstrated by the attention researchers pay to the psychological side. This is shown in a Dutch study with a group of 300 patients who were confirmed to have pSS. They completed several validated questionnaires assessing fatigue, perception of physical activity, perception of illness, cognitive regulation, emotion processing and regulation, coping strategies, and social support.

After conducting interviews with the patients, four psychological profiles were identified: functional, alexithymic, independent, and dysfunctional. For patients with all these profiles, fatigue was higher than in the general population and had an influence on personality type [28].

### 1.2. Objectives

The present work aims to translate two questionnaires, Profile of Fatigue and Discomfort–Sicca Symptoms Inventory (PROFAD-SSI-SF) and Primary Sjogren’s Syndrome—Quality of Life (PSS-QoL), from English into Romanian, and culturally adapt and validate them in order to assess the fatigue and quality of life of patients with Sjogren’s syndrome. This choice is justified by the fact that there is no validated instrument in Romanian to assess fatigue and quality of life in patients with Sjogren’s syndrome.

## 2. Materials and Methods

The translation and cultural adaptation of the two questionnaires for the Romanian context were performed according to the guidelines accepted at the international level and recommended by the International Society of Pharmacoeconomics and Research (ISPOR), the data from the international literature, and the recommendations of the World Health Organization (WHO) regarding the translation process and adaptation instruments [29,30].

**Figure 1:**
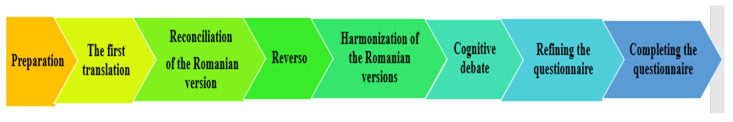
Overview of the steps required for the translation and validation of the questionnaires.

The two translated questionnaires used in this study can be found in Appendix A. Several steps were required in the process of translation and validation, which are summarized in Figure 1.

### 2.1. Description of the Translation Process

The conceptual, semantic, and operational equivalence of PROFAD-SSI and PSS-QoL complied with international guidelines for the translation, adaptation, and validation of the questionnaires and included the following phases: preparation, first translation, the tuning of the Romanian version, Reverso, the harmonization of the Romanian versions, cognitive debate, the refinement of the questionnaire, and the finalization of the final form of the questionnaires. The working group responsible for the development of these assessment tools included eight doctors and a translator, and all members of the group were bilingual in Romanian and English.

The questionnaires were completed by patients with primary Sjogren’s syndrome diagnosed by the treating rheumatologist according to the 2016 ACR-EULAR classification criteria. Patients had to be at least 18 years old and gave their consent to participate by signing the informed consent form.

The translation process was carried out systematically and comprised several stages:

First, permission for translation was obtained from the two main authors of the questionnaires [17,18]. Subsequently, the protocol and informed consent forms for the patients were developed, based on which approval was obtained from the Ethics Committee of the Sfanta Maria Clinic Hospital Bucharest. This phase also included the recruitment and training of translators (e.g., using the conceptual translation of a word or phrase; providing a simple, clean, and concise version; and avoiding jargon or terms that cannot be understood by ordinary people in everyday life, regardless of their age, education, and culture), followed by the development of independent translations from English into Romanian, carried out by members of the working team and an independent translator who is proficient in English but not a native speaker.

The next phase consisted of the back-translation of the agreed Romanian version into English (carried out by an independent translator, a native English speaker who is fluent in Romanian and had nothing to do with the questionnaire). The purpose was to ensure the quality of the translation and confirm that the original meaning of each question in the questionnaire was preserved.

Thus, a harmonization procedure of the previous Romanian versions of the questionnaires was carried out by comparing them with the original English version. This process resulted in a second version of the questionnaires in Romanian.

This final version of the two questionnaires was administered to a group of 27 patients with primary Sjogren’s syndrome, after which a third version of the questionnaire was created.

This final version of the questionnaire was the result of adaptations made based on feedback and observations from the first group of patients. These adjustments were aimed at clarifying terms, refining the wording, and ensuring that the questions were clearly understood.

The version obtained in this way was then given to a group of 25 patients using the methodology mentioned above. This resulted in the final versions of the two questionnaires in Romanian: PROFAD-SSI-SF and PSS QoL.

The questionnaires were handed out in the semantic equivalence assessment phases in a face-to-face meeting. This method of administering the questionnaires had no influence on the use of the self-completed questionnaire. During completion, the patient’s understanding, recognition, and judgment were analyzed.

The decision to use face-to-face questionnaires was also supported by the advantages offered by this method: its applicability to all age groups, the ability of interviewers to ensure respondents’ attention without distraction, and the avoidance of excluding patients with eye problems.

Following each phase, inappropriate translation terminology was identified and amended, and any inconsistencies between the previous translation and the current versions were resolved. Discussions were also held to clarify word meanings, and alternatives were proposed until a consensus was reached.

The data obtained from the patients were entered into a database and statistically analyzed using SPSS version 29.0.1.0 (171) (© copyright TBM Corporation and its licensors 1989, 2023).

### 2.2. Data on PROFAD-SSI-SF (Profile of Fatigue and Discomfort–Sicca Symptoms Inventory—Short Form) and PSS-QoL (Primary Sjogren’s Syndrome—Quality of Life Questionnaire)

Recent studies have shown that for a complete assessment of a patient with Sjogren’s syndrome, it is necessary to determine saliva [31,32,33] and tear production [34,35,36] but also to evaluate fatigue, quality of life, and the consequences caused by the systemic manifestations [37,38]. Reliable assessment tools are necessary to ensure both the accuracy of the assessment and its usability in clinical trials.

In order to have tools to assess fatigue and quality of life in Romanian patients with Sjogren’s syndrome, we proposed the translation and validation of the following two questionnaires: PROFAD-SSI-SF and PSS-QoL. The first questionnaire has passed the test of time and has proved to be a complex evaluation tool, and the second questionnaire is the only one specifically addressed to patients with primary Sjogren’s syndrome.

### 2.3. PROFAD-SSI-SF: Short History

The PROF questionnaire, developed in 2004 by Bowman et al., is intended to assess fatigue in individuals suffering from rheumatoid arthritis, systemic lupus erythematosus, and primary Sjogren’s syndrome. Its utility was illustrated by comparisons with other well-known scales, including the SF-36, WHOQOL-BREF, and HAD [15]. A longer version of this survey, known as the PROFAD-SSI questionnaire, was later released in the same year. There are eight areas of disease activity in this 64-item patient-reported outcome measure: two somatic and mental fatigue symptoms and a general discomfort question including arthralgia, vascular dysfunction, mouth dryness, ocular dryness, skin, and vaginal dryness.

After the administration of the questionnaire, a classification of mental fatigue into ‘poor concentration’ and ‘impaired memory’ was developed, raising five hypotheses about the causes of fatigue: one mental cause and four somatic causes.

Structurally, the questionnaire can be described as consisting of items related to mental and somatic fatigue, contributing to the fatigue profile (PROF). Scoring for arthralgias, vascular dysfunction, skin issues, eye problems, and oral symptoms (PROFAD) are also included, as well as Sicca symptomatology (PROFAD-SSI).

The development of the PROFAD-SSI questionnaire began with the premise that patients’ personal descriptions of symptoms are the most reliable sources for evaluation [39]. Therefore, responses are quantified using a Likert scale ranging from 0 to 7 points, where 0 represents ‘no problems’ and 7 signifies ‘the worst imaginable’ symptoms experienced by the patient.

Subsequently, in 2009, a simplified version of this questionnaire with 19 questions, all belonging to the same eight domains, was created and validated for ease of use in clinical trials [15,17].

Considering the significance of this questionnaire, supported by its correlation with disease activity and comparison with ESSPRI, an international index for assessing the symptoms of primary Sjogren’s syndrome patients, we believe it is essential to validate this tool for the Romanian context. It can serve both research purposes and applications in the daily clinical practice of patients [40].

### 2.4. PSS-QoL: Short History

Over time, several patient-reported outcomes (PROs) have been developed to assess patients with Sjogren’s syndrome, which have also involved application in clinical trials. Two of these PROs are PROFAD-SSI and ESSPRI. However, none of these measures were specifically designed to evaluate the physical and social consequences of the disease’s clinical manifestations. For example, individuals who experience dry mouth may encounter speech difficulties, leading to psychological and emotional challenges, difficulties at work, and a diminished social life [41,42].

In numerous clinical trials involving patients with Sjogren’s syndrome, various well-established quality-of-life assessment scales have been utilized, such as SF-36, Euro-Qol-5D, or HADS. In 2017, the first questionnaire dedicated to patients with Sjogren’s syndrome, known as PSS-QoL, was validated. This questionnaire aims to assess the disease not only from a clinical perspective but also in terms of its social impact and its influence on quality of life [43].

The questionnaire is divided into two sections for evaluation: a clinical assessment section and a psychosocial evaluation section. It consists of 25 questions, referring to the four weeks before the evaluation. Importantly, one of the questions is exclusively directed to women, and the total score of the questionnaire ranges from 0 to 96 for women and 0 to 92 for men [18].

A numerical scale is employed to assess the physical domain (specifically pain), while yes/no questions are used to evaluate systemic manifestations, providing further details regarding sensory pathology if an affirmative response is given. The psychosocial domain is evaluated using 14 questions, with responses ranked on a Likert scale from 0 to 5. The validation of this questionnaire demonstrated a robust correlation between the individual items and the overall test score. The development strategy employed was similar to that of PROs such as PSAID for psoriatic arthritis or RA-Qol for rheumatoid arthritis. Therefore, the primary significance of PSS-QoL lies in its ability to assess not only the clinical manifestations but also the social impact and the effect on the quality of life of individuals with Sjogren’s syndrome. This encompasses various aspects such as self-confidence, fatigue, and emotional burden [22].

## 3. Results

Two groups of patients were used for the translation and validation of the questionnaires, primarily comprising females with an average age of 48.25 (±12.03) years. These findings align with the existing research, which indicates an estimated female-to-male ratio of 14–9 to 1 [44]. The majority of patients originate from urban environments, accounting for 67.3% of the sample. In terms of education, most patients have completed the middle–high school education cycle, representing 40.4%. Concerning the duration of patients’ illness, the average duration was 7.11 (±5.25) years.

All demographic characteristics of the patients are listed in Table 1.

The choice to translate the two questionnaires (PROFAD-SSI-SF and PPS-QoL) is justified by the fact that they are complementary, with the goal of not only conducting a clinical assessment but also assessing the social impact caused by Sjogren’s syndrome. A more thorough understanding of the consequences determined by dryness and systemic manifestations will help in adopting a more effective therapeutic approach.

The initial stage consisted of translation and cultural adaptation. During this stage, regarding the PROFAD-SSI questionnaire, there were no difficulties in understanding. However, two patients suggested replacing the word ‘fatigabilitate’ with the word ‘oboseala’, justifying it with a clearer and easier understanding. In response, the translation team agreed to add the explanation of the word ‘fatigabilitate’ in parentheses, as it has a complex meaning and could not be completely removed.

Regarding the translation and cultural adaptation of the second questionnaire, PSS-QoL, during the evaluation, patients made specific observations, which will be described below. This questionnaire provides various response formats. Thus, for the first question, a numerical scale is used for the answer, followed by 10 yes/no questions, and the subsequent questions allow respondents to quantify their answers based on the frequency of described symptomatic episodes.

For Question 1, ‘How severe was your pain?’, one patient suggested that the term ‘pain’ should be more specific and indicate the area affected as it is a broad term. Another patient referred to joint pain when answering this question. In response, one patient suggested replacing the term ‘durere’ with ‘suferinta’. The translation team decided to retain the term ‘durere’, which encompasses all types of pain experienced by patients, while ‘suferinta’ has a more emotional connotation stemming from ‘pain’.

For Question 3, ‘I had recurrent walking pain’, one patient suggested adding the description of the type of pain, namely ‘articular’. The research team decided not to use the description of the type of pain or location as this concept refers to all types of pain the patient may have.

For Question 8, ‘Do your eyes feel dry?’, the patient had to choose between ‘no’ and ‘yes’, and in the case of a positive answer, several manifestations of dry eyes were indicated. In this case, one patient suggested changing the translation ‘fara lacrimi’ (without tears) to ‘lipsa lacrimilor’ (lack of tears), as the original statement was ‘no tears’. The research team agreed to change the translation.

For Question 10, in which patients are asked ‘Does your nose feel dry?’, the patient had to select ‘change in sense of smell’ or ‘nosebleed’ as the clinical manifestation in the case of a positive answer. In this case, three patients identified with the feeling of a dry nose, but they had none of the mentioned manifestations. The research team decided to keep the original version as the same situation could be present, so the patient does not have to tick any of the manifestations.

For Question 11, ‘Does your vagina feel dry?’, as in the case of the previous question, one patient answered affirmatively but did not fit into any of the clinical manifestations mentioned in the question. Another patient suggested adding a subsection titled ‘otherwise’, where patients can freely fill in their responses. Since associating a section would change the question and topic, the research team decided to keep the current question.

Following a critical evaluation of patient observations and interpretation of modification proposals in the questionnaire’s context, the final version of the two questionnaires was generated.

The second stage involved validating the two questionnaires. For the pilot study, 27 patients initially completed them, and later, the questionnaires were retested on 25 patients. The number of patients was established in accordance with international guidelines for translating and validating a questionnaire.

The reliability of the questionnaires was determined by the consistency of patients’ answers; thus, we measured the internal consistency for both questionnaires [45]. A Cronbach alpha index of 0.83 was obtained for PROFAD-SSI-SF, and a Cronbach alpha index of 0.93 was obtained for PSS-QoL. These findings also demonstrate a strong correlation between each item and the overall score for questionnaires translated into the Romanian language.

The reliability of both questionnaires was firmly established, as demonstrated by the highly significant statistical results obtained from the interclass correlation coefficient (ICC). For the PPS-QOL, the ICC yielded an impressive average measure of 0.971, with a 95% confidence interval (CI) ranging from 0.958 to 0.981. These findings unequivocally confirm the excellent reliability of the PPS-QOL instrument. Similarly, the ICC for PROFAD-SSI-SF yielded an outstanding average measure of 0.986, with a 95% confidence interval (CI) ranging from 0.980 to 0.991. These results leave no room for doubt regarding the exceptional reliability of the PROFAD-SSI-SF instrument (see Table 2).

For the psychosocial part of the PSS-QoL, principal component analysis (PCA) was conducted in this study. This multivariate statistical technique allows researchers to examine the underlying structure and relationships within a dataset. The decision to proceed with PCA was supported by the robustness of the data as indicated by a high Kaiser–Meyer–Olkin (KMO) measure of sampling adequacy with a value of 0.913. Bartlett’s test for sphericity, a significance test used to check whether the correlations between the variables are sufficiently different from zero, also yielded a highly significant result with *p* < 0.001, confirming the suitability of PCA for the dataset.

In addition, all individual items had significant nonrotating factor weights, each exceeding the threshold of 0.3. These factor weights provide information about the contribution of each item to the principal component determined by PCA. The values ranged from 0.898 to 0.966, emphasizing the strong relationships and shared variance between the items within the dataset. The presence of such high factor weights for a single component indicates a one-dimensional structure within the data, which emphasizes the applicability of PCA for this analysis (see Table 3).

## 4. Discussion

This study represents the first translation and cultural adaptation into Romanian of two questionnaires used to assess quality of life and fatigue in patients with Sjogren’s syndrome, as well as clinical manifestations. Currently, there is no questionnaire translated and validated for the Romanian language that allows a complex assessment of patients with Sjogren’s syndrome.

The results of the current study showed limited variability in the interpretation of the items and good feasibility for both questionnaires. Although patients’ ability to understand and communicate may be influenced by factors such as age, education, and clinical manifestations related to the disease (e.g., eye damage or ‘brain fog’), no significant differences in patients’ responses based on these variables were found in our study.

Although the main goal of therapy is to improve clinical symptoms as well as paraclinical parameters, there has been an upward trend in the treatment of fatigue in recent years. This multifaceted approach is primarily aimed at improving quality of life [46,47].

Analyzing patients’ quality of life gives us a better understanding of the consequences of disease and treatment and provides us with a better insight into medical decision making. At the same time, it is an important goal in medical research and the healthcare industry [48]. The best assessment methods are questionnaires that include numerical scales or visual analog scales, allowing for the easy interpretation of the results [49].

The PSS-QoL also belongs to this category. In 2021, Lackner A et al. published a study showing that patients’ perception of dryness assessed with the PSS-QoL correlated with objective measures of salivary function, whereas this was not the case with the ESSPRI. As a result, two categories of patients were identified: one group with a high perception of dryness and impaired quality of life and a second group with a lower perception of dryness but higher clinical and biological activity. It is important to note that this questionnaire assesses all affected areas [19].

The most important limitation of this study is the small size of the participants. Another limitation is the lack of an assessment of the sensitivity of the questionnaires at different stages of patients’ disease progression, especially after treatment administration. We, therefore, recommend expanding the study in the future using a larger patient cohort and a wider range of distribution methods to fully demonstrate the psychometric properties of the questionnaires.

## 5. Conclusions

This pilot study reveals that the Romanian version of the PROFAD-SSI and PSS-QoL questionnaires is as reliable as their English counterparts.

Consequently, the Romanian adaptations of the two questionnaires for the assessment of quality of life and fatigue in patients with primary Sjogren’s syndrome can be used both for research purposes and as a new assessment tool in daily clinical practice. Now that a valid instrument is available to quantify the impact of the disease, new perspectives can emerge in the management of these patients, and its continued use will further consolidate its robustness.

It is important to emphasize a notable limitation of this study, namely its relatively small sample size, which is a typical feature of a pilot study. The deliberate decision to start with a limited number of patients is in the nature of pilot studies, which are primarily concerned with testing feasibility, methodology, and preliminary results that, while providing valuable insights and a basis for future research, should be considered indicative rather than conclusive.

The limitations inherent in this study serve as a stepping stone that paves the way for later validation and expansion of our research to a larger and more diverse cohort of patients. With this preliminary study, we have laid the groundwork for future studies that will include a broader range of participants and provide a more comprehensive understanding of the topic.

## Figures and Tables

**Table 1:** Demographic characteristics of patients (*n* = 52).

	*n*	%
Sex	50	87.7
Women		
Men	2	3.5
Age (mean ± SD, years)	48.25 ± 12.03	
20–30	5	9.61
31–40	8	15.38
41–50	17	32.69
51–60	14	26.92
61–70	8	15.38
Urban Dwelling	35	67.3
Native Romanian Speaker	52	100
Education		
Higher (above high school level)	19	36.53
High or assimilated	23	44.23
Basic	10	19.23
Duration of Disease (mean ± SD, years)	7.11 ± 5.25	

**Table 2:** Interclass correlation coefficient for PROFAD-SSI-SF and PSS-QoL questionnaires.

	Interclass Correlation	95% Confidence Interval Lower Bound Upper Bound	
Single measures for PROFAD-SSI-SF	0.790	0.722	0.854
Average measures for PROFAD-SSI-SF	0.986	0.980	0.991
Single measures for PSS-QoL	0.598	0.475	0.673
Average measures for PSS-QoL	0.971	0.958	0.981

**Table 3:** Component matrix. Extraction method: principal component analysis; one component extracted.

	Factor Weights
I have a feeling that I am the only person with these complaints	0.935
I have a feeling that my complaints are not taken seriously	0.934
I have a feeling that my complaints are too much for me	0.959
I have a feeling that my family and friends are understanding	0.908
I am too tired to fulfill obligations to my family and friends	0.951
I am withdrawn	0.938
I am concerned about the side effects	0.898
I worry about the further course of my disease	0.925
I have a good feeling about my body	0.944
I cannot manage my everyday life as well as I did before I became ill	0.966
I tire easy	0.964
Everyday activities like driving, work, household, and sports are a challenge	0.940
Remedies like eye drops, creams, and physiotherapy impose a financial burden	0.933
The disease has reduced my quality of life	0.912

## Data Availability

Please contact the corresponding author for any inquiries regarding data access.

## Appendix A

**Profilul de fatigabilitate (oboseala) si disconfort in randul pacientilor cu sindrom Sjogren si chestionar simptome Sicca-forma scurta(PROFAD-SSI-SF) in limba romana**

*Va rugam sa evaluati cat de sever a fost fiecare simptom in ultimele 2 saptamani selectand unul din numerele de la 0 la 7*

1. In ultimele 2 saptamani cea mai grava problema pe care am avut-o in ceea ce priveste nevoia de odihna, senzatia de oboseala, epuizare sau nevoia de somn a fost:

Nu am avut nevoie sa ma odihnesc deloc 0 1 2 3 4 5 6 7 cea mai grava posibil

2. In ultimele saptamani cat de dificil a fost sa incep o activitate ce presupunea un efort sau imi dadea senzatia ca duc o batalie:

Deloc dificil 0 1 2 3 4 5 6 7 cel mai dificil posibil

3. In ultimele 2 saptamani cat de dificila a fost mentinerea unei activitati a ca urmare a pierderii rapide a energiei sau ramanerii fara vlaga:

Deloc dificil 0 1 2 3 4 5 6 7 cel mai dificil posibil

4. In ultimele 2 saptamani cea mai mare problema legata de lipsa de forta in muschi sau senzatia de slabiciune a fost:

Fara slabiciune musculara 0 1 2 3 4 5 6 7 cel mai rau de imaginat

5. In ultimele 2 saptamani cea mai grava problema legata de gandirea neclara sau lipsa concentrarii a fost:

Fara astfel de probleme 0 1 2 3 4 5 6 7 cel mai rau imaginabil

6. In ultimele 2 saptamani cea mai grava problema legata de uitarea ideilor sau greseli frecvente a fost:

Fara astfel de probleme 0 1 2 3 4 5 6 7 cel mai rau imaginabil

7. In ultimele 2 saptamani cea mai dificila problema legata de disconfortul de la nivelul membrelor (de ex disconfort sau dureri in articulatiilor mari-sold, genunchi, umeri) in muschi sau durere generalizata a fost:

Fara astfel de probleme 0 1 2 3 4 5 6 7 cel mai rau imaginabil

8. In ultimele 2 saptamani cea mai dificila problema legata de disconfortul sau umflarea degetelor sau incheieturilor mainilor a fost:

Fara astfel de probleme 0 1 2 3 4 5 6 7 cel mai rau imaginabil

9. In ultimele 2 saptamani cea mai dificila problema legata de disconfortul dat de manilie reci a fost:

Fara astfel de probleme 0 1 2 3 4 5 6 7 cel mai rau imaginabil

10. In ultimele 2 saptamani cea mai dificila problema legata de pielea uscata sau mancarime a pielii a fost:

Fara astfel de probleme 0 1 2 3 4 5 6 7 cel mai rau imaginabil

11. In ultimele 2 saptamani cea mai dificila problema legata de uscaciunea vaginala (de exemplu. am experimentat disconfort în timpul actului sexual din cauza uscăciunii vaginale) a fost:

Fara astfel de probleme 0 1 2 3 4 5 6 7 cel mai rau imaginabil

12. In ultimele 2 saptamani cea mai dificila problema legata de durerea oculara (de exemplu: senzatie de nisip in ochi, ochi durerosi, senzatie de arsura, mancarime sau iritatie oculara) a fost

Fara astfel de probleme 0 1 2 3 4 5 6 7 cel mai rau imaginabil

13. In ultimele 2 saptamani cea mai dificila problema legata de iritatia ochilor (de exemplu: ochi iritati de atmosfera poluata/vant/aer conditionat/umiditate scazuta) a fost:

Fara astfel de probleme 0 1 2 3 4 5 6 7 cel mai rau imaginabil

14. In ultimele 2 saptamani cea mai dificila problema legata de vederea slaba chiar purtand ochelari (de exemplu, vedere încețoșată, slabă, probleme la citire, probleme la vizionarea la televizor sau conducerea nocturnă, ecranul computerului sau ecranul bancomatului) a fost:

Fara astfel de probleme 0 1 2 3 4 5 6 7 cel mai rau imaginabil

15. In ultimele 2 saptamani cea mai dificila problema legata de dificultatea de a mânca (de exemplu: senzatie de gura uscata, dificultate la înghițit, nevoie de a consuma lichide pentru a înghiți alimente/alimente lipite in gură, nevoia de clătire a resturilor alimentare sau gust alterat) a fost:

Fara astfel de probleme 0 1 2 3 4 5 6 7 cel mai rau imaginabil

16. In ultimele 2 saptamani cea mai dificila problema legata de uscaciunea gâtului sau nasului (de exemplu: gura uscata la respiratie, dificultate de a vorbi, nevoia de a bea lichide pentru usurarea vorbirii, nas uscat, senzatie de gât uscat, gura uscată in aer condiționat) a fost:

Fara astfel de probleme 0 1 2 3 4 5 6 7 cel mai rau imaginabil

17. In ultimele 2 saptamani cea mai dificila problema legata de mirosul respiratiei (de exemplu respiratie urat mirositoare/saliva lipicioasă) a fost:

Fara astfel de probleme 0 1 2 3 4 5 6 7 cel mai rau imaginabil

18. In ultimele 2 saptamani cea mai mare problema legata de nevoia de a-ti umezi gura: de exemplu. sa iti aduci apa de baut la pat, senzatie de sete pe timpul noptii, urinare frecventa nocturna, senzatie urgenta de a urina a fost:

Fara astfel de probleme 0 1 2 3 4 5 6 7 cel mai rau imaginabil

19. In ultimele 2 saptamani cea mai mare problema legata de alte suferinte la nivelul gurii (de exemplu. ulcere bucale, glandele salivare umflate, senzatie de sufocare datorita uscăciunii, modificării ale aromelor sau gusturilor, necesitatea unui consult stomatologic) a fost:

Fara astfel de probleme 0 1 2 3 4 5 6 7 cel mai rau imaginabil

**Chestionar privind calitatea vietii pacientilor cu sindrom Sjogren primar (PSS-QoL) in limba romana**

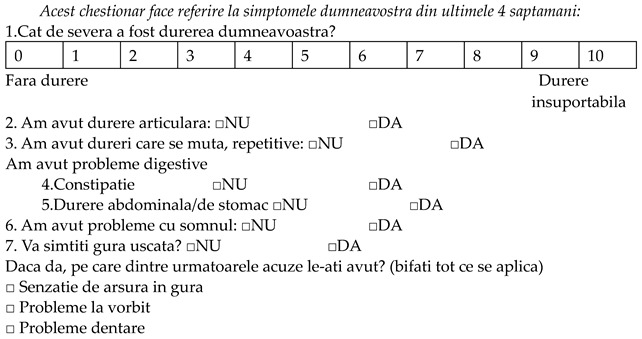

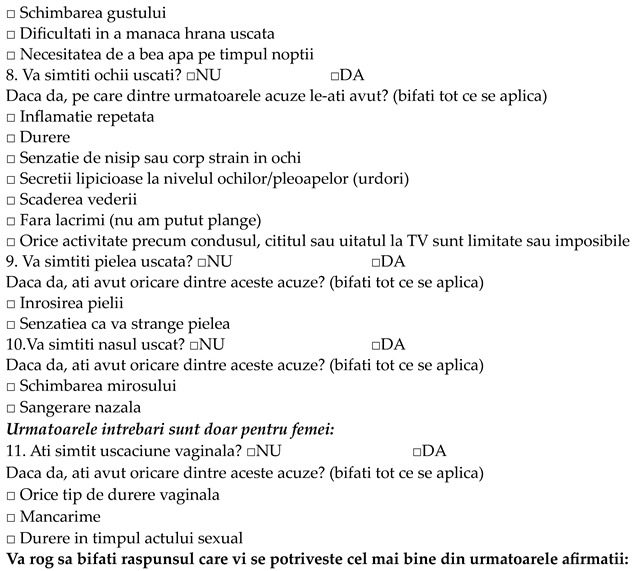

	Niciodata	Rar	Cateodata/Uneori	Deseori	Mereu
Am senzatia ca: 12. Sunt singura persoana cu astfel de acuze 13. Acuzele mele nu sunt luate in serios 14. Acuzele mele ma coplesesc 15. Familia si prietenii mei sunt intelegatori					
16. Sunt prea obosit/a sa imi indeplinesc obligatiile fata de familie si prieteni					
17. Sunt retras/a					
18. Sunt ingrijorat/a de efectele adverse					
19. Sunt ingrijorat/a de evolutia viitoare a bolii mele					
20. Ma simt bine in corpul meu					
21. Nu imi pot organiza viata de zi cu zi asa cum o faceam inainte sa ma imbolnavesc					
22. Obosesc usor					
23. Orice activitate precum sofatul, serviciul, intretinerea casei sau sportul reprezinta o provocare					
24. Remediile precum lacrimile artificiale, cremele si fizioterapia reprezinta o povara finanicara					
25. Aceasta boala mi-a scazut calitatea vietii					
